# Impact of Casein Phosphopeptide Amorphous Calcium Phosphate and Proanthocyanidin on Bond Strength of Universal Adhesives to Caries‐Affected Dentin in Primary Teeth: An In Vitro Study

**DOI:** 10.1002/cre2.70131

**Published:** 2025-04-22

**Authors:** Ali Nozari, Farnaz Haji Abbas Oghli, Fatemeh Parvizi, Zahra Jowkar, Maryam Pakniyat Jahromi, Seyed Ahmadreza Hamidi

**Affiliations:** ^1^ Department of Pediatric Dentistry, School of Dentistry Shiraz University of Medical Sciences Shiraz Iran; ^2^ Oral and Dental Disease Research Center, Department of Operative Dentistry School of Dentistry, Shiraz University of Medical Sciences Shiraz Iran; ^3^ Department of Oral and Maxillofacial Surgery, School of Dentistry Shiraz University of Medical Sciences Shiraz Iran

**Keywords:** caries‐affected dentin, casein phosphopeptide‐amorphous calcium phosphate, microshear bond strength, primary teeth, proanthocyanidin

## Abstract

**Objectives:**

This study aimed to assess the impact of casein phosphopeptide‐amorphous calcium phosphate (CPP‐ACP) and proanthocyanidin (PA) on the microshear bond strength (μSBS) of universal adhesives to caries‐affected dentin (CAD) in primary teeth.

**Materials and Methods:**

160 human primary second molars with occlusal caries were utilized, with CAD‐exposed dentin surfaces. The teeth were categorized into four groups based on CAD pretreatment: no pretreatment, CPP‐ACP for 3 min, PA for 1 min, and PA for 1 min followed by CPP‐ACP for 3 min. Each group subdivided into four based on adhesive system (Gluma Bond Universal or All‐Bond Universal) and application mode (etch and rinse; E&R or self‐etch; SE). Following composite resin restoration, μSBS measurements were taken after 24 h of water storage.

**Results:**

PA pretreatment showed the highest μSBS compared to controls and other methods (*p* < 0.001). Conversely, CAD pretreatment with CPP‐ACP + PA led to lower μSBS than the control (*p* = 0.009). Universal adhesive choice significantly influenced μSBS (*p* < 0.001), with Gluma Bond Universal outperforming All‐Bond Universal (*p* < 0.001). The E&R method demonstrated superior bond strength over SE (*p* < 0.001).

**Conclusion:**

CAD pretreatment, particularly with PA, significantly impacted bond strength, with Gluma Bond Universal and the E&R method proving optimal for enhancing μSBS to CAD. These findings offer valuable insights for refining adhesive protocols in pediatric dentistry, potentially improving clinical outcomes in restorative procedures.

## 1 Introduction

Currently, dental practitioners strive to eliminate infected dentin while preserving caries‐affected dentin (CAD) within the cavity (Jowkar et al. 2021). The cyclic process of demineralization and remineralization in CAD results in reduced mineral content and increased porosity in the intertubular dentin, compared to normal dentin (Lenzi et al. 2015). These factors contribute to diminished bond strength and an increased occurrence of exposed collagen fibrils within the hybrid layers, unlike in sound dentin. Over time, these conditions can lead to the degradation of unprotected collagen due to the action of bacterial and host‐mediated enzymes (Marquezan et al. 2010).

Various efforts have been made to promote remineralization of the porous and hypomineralized intertubular dentin in CAD (Jowkar et al. 2021). One approach involves applying a paste containing casein phosphopeptide‐amorphous calcium phosphate (CPP–ACP), such as MI paste (GC Corporation, Tokyo, Japan), which creates a supersaturated state for bioavailable calcium and phosphate ions in the dental substrate (Jowkar et al. 2021). This allows for the diffusion of calcium and phosphate ions into the porous lesion, leading to the deposition and remineralization of partially demineralized crystals (Poggio et al. 2013).

While CPP‐ACP has been discussed for its role in CAD remineralization, proanthocyanidin (PA) emerges as another promising candidate. PA has shown potential in facilitating the remineralization of CAD by promoting the deposition of vital minerals, such as calcium and phosphate, essential for strengthening the tooth structure (Beckman et al. 2024). PA, a polyphenolic compound, acts as a potent antioxidant (Beckman et al. 2024). PA has been associated with increased collagen synthesis and reduced enzymatic degradation of collagen matrices (Beckman et al. 2024). Remarkably, PA has demonstrated exceptional cross‐linking effectiveness within short treatment durations (Liu and Wang 2013). Additionally, PA exhibits superior inhibitory effects on endogenous matrix metalloproteinases (MMPs) to chlorhexidine (Epasinghe et al. 2013).

Recent advancements in adhesive systems have introduced universal adhesives, also known as “multi‐mode” or “multi‐purpose” adhesives, consolidating all essential bonding components into a single bottle (Lenzi et al. 2015). These versatile adhesives can be employed in both etch‐and‐rinse (E&R) or self‐etch (SE) modes, offering flexibility in application (Lenzi et al. 2017). Studies on universal adhesives in primary teeth are limited but have shown varying outcomes. While one study noted lower bond strength to primary dentin with a SE approach using Scotchbond Universal Adhesive, another study demonstrated enhanced bond strength to primary tooth dentin with Scotchbond Universal and All‐Bond Universal following prior acid etching (Kim et al. 2017; Lenzi et al. 2017). Nicoloso et al. found no significant disparity in bond strength between SE and E&R methods (Ferreira Nicoloso 2016). These investigations have primarily focused on intact dentin, aligning with modern restorative dentistry's emphasis on ultraconservative approaches for carious lesions, which aim to preserve and potentially remineralize affected dentin within cavity preparations (Lenzi et al. 2015). Consequently, ongoing discussions persist regarding the influence of application modes on the bond strength of universal adhesives to primary teeth (Lenzi et al. 2013; Ferreira Nicoloso 2016; Thanaratikul et al. 2016, Kim et al. 2017).

Preserving the integrity of dentin collagen within the hybrid layer of CAD is paramount for successful restorative procedures. The combined effects of CPP‐ACP's remineralization properties and PA's anticollagenolytic and crosslinking capabilities may play a crucial role in maintaining this integrity. However, a comprehensive understanding of how these agents impact the bonding performance of universal adhesives in primary teeth affected by caries is lacking. Therefore, this in vitro study aims to address this gap by evaluating the micro‐shear bond strength (μSBS) of two universal adhesive systems applied with CPP‐ACP and PA on CAD in primary teeth. The study aimed to test three null hypotheses: (a) the pretreatments of the dentin with CPP‐ACP or PA have no impact on the bonding performance of the universal adhesive, (b) there is no significant difference in bond strength between the two universal adhesives in CAD of primary teeth, (c) the universal adhesives exhibit similar bonding performance to primary dentin regardless of the etching technique.

## 2 Methods and Materials

### 2.1 Specimen Preparation

The study acquired a collection of one hundred and sixty human primary second molars, which had occlusal caries extending approximately halfway into the dentin. The identification of occlusal caries extending approximately halfway into the dentin was conducted using clinical and radiographic evaluation. The teeth were first examined visually and tactilely using a dental explorer to assess surface characteristics. Additionally, periapical radiographs were taken to confirm the depth of the carious lesions. Only teeth with caries reaching about halfway into the dentin, as determined by radiographic examination, were included in the study. These teeth were obtained with the authorization of the Research and Ethics Committee of Shiraz University of Medical Sciences, and they had been extracted for orthodontic purposes (Protocol #IR.SUMS.DENTAL.REC.1402.056). The parents of the patients were fully informed about the study's purpose and the use of the extracted teeth. They provided consent by signing relevant forms. An experienced operator, unaware of the study conditions, performed all experimental procedures. The collected teeth were cleaned using a periodontal curette and stored in a 0.5% chloramine T solution at 4°C. The storage period did not exceed 1 month before the teeth were utilized for the study.

The sample size for the experiment was determined using G Power software (G∗Power 3.1 software; Heinrich Hein University, Dusseldorf, Germany) before the commencement of the study. The calculation of the sample size was based on a comparison between two means ( ± standard deviations) obtained from a previous research, where the means were 21.86 ± 1.6 and 24.44 ± 1.6, and the standard deviations were 1.6 for both groups (Jowkar et al. 2021). With a type I error (α) set at 0.05 and a desired power level of 80%, the analysis indicated that a minimum of seven specimens per subgroup would be necessary for the current study. However, in recognition of the anticipated dropout rate of approximately 25% and the need to account for this potential dropout, the sample size was adjusted to 10 teeth per subgroup. This adjustment was made to ensure a sufficient number of teeth remain in each subgroup, accounting for potential data loss and maintaining the statistical power of the study.

To expose flat midcoronal dentin surfaces perpendicular to the teeth's long axis, the occlusal enamel and superficial dentin of the crown were removed using a water‐cooled low‐speed cutting machine (Mecatome T201 A, Presi, Grenoble, France). Following that, the Caries Detector (Kuraray Co, Tokyo, Japan) was used in accordance with the manufacturer's instructions. Various criteria, including hardness to the sharp excavator, visual examination, and staining with dye, were employed to differentiate between caries‐affected and normal dentin. The removal of infected dentin was repeated for each tooth until only the discolored, harder dentin that stained pink remained, which was considered as CAD (Jowkar et al. 2021). Wet 600‐grit SiC paper was utilized for additional finishing, carried out for 20 s under running water. After sectioning the roots 1 mm below the cementoenamel junction, the samples were embedded in acrylic resin (Acropars; Marlik Co., Tehran, Iran) with the dentin surfaces positioned parallel to the bottom of the mold.

### 2.2 Experimental Groups

**Figure 1:**
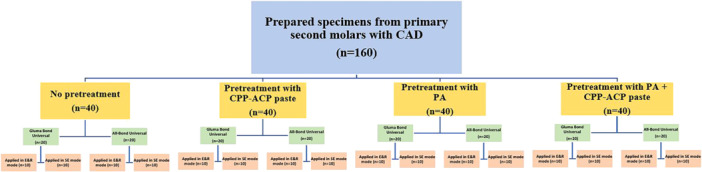
Diagram illustrating the study design. CAD, Caries‐affected dentin; CPP‐ACP paste, casein phosphopeptide‐amorphous calcium phosphate paste; E&R, etch and rinse; PA, proanthocyanidin; SE, self‐etch.

Figure 1 provides a visual representation of the study groups along with their corresponding study protocols. The materials utilized in this study are presented in a comprehensive manner in Table 1.

**Table 1:** Materials specifications.

Material	Type	Manufacturer	Composition	Application procedure
Gluma Bond Universal	Universal adhesive bonding system	Heraeus Kulzer GmbH, Hanau, Germany	MDP phosphate monomer, 4‐META, dimethacrylate resins, acetone, fillers, initiators, silane	SE mode: 1. Apply the adhesive to the entire prepared surface with the applicator brush and rubbed for 20 s 2. Dry sufficiently by blowing mild air for more than 5 s until the adhesive resin does not move 3. Light‐cure for 10 s Etch and rinse mode: 1. For enamel specimens, apply Phosphoric acid etching gel (37%) to enamel and left in place for 20 s; for dentin specimens, apply Phosphoric acid etching gel (37%) to dentin and left in place for 15 s 2. Rinse the prepared surface using air‐water spray for 30 s and dried with absorbent paper 3. Apply the adhesive to the entire prepared surface with the applicator brush and rubbed for 20 s 4. Dry sufficiently by blowing mild air for more than 5 s until the adhesive resin does not move 5. Light‐cure for 10 s
All Bond Universal	Universal adhesive bonding system	Bisco; Schaumburg, IL, USA	Bis‐GMA, 10‐MDP, HEMA, ethanol, initiators, water	Self‐etch mode: 1. Dispense 1‐2 drops into a clean well. 2. Apply two separate coats, scrub the preparation with a microbrush for 10–15 s per coat. Do not light cure between coats. 3. Evaporate excess solvent by thoroughly air drying with an air syringe for at least 10 s; there should be no visible movement of the adhesive. The surface should have a uniform glossy appearance. 4. Light cure for 10 s. Etch and rinse mode: 1. Etch dentin using an etchant for 15 s. Rinse thoroughly. Remove excess water by blotting the surface with an absorbent pellet or high‐volume evacuation for 1–2 s, leaving the preparation visibly moist. 2. Apply adhesive as self‐etch technique.
Filtek^MR^ Z350 XT	Composite resin	3M‐ESPE, St Paul, USA.	Ceramics treated with Silane, BIS‐GMA, BIS‐silane‐treated silica, EMA, silicazirconia oxide treated with Silane, diuretano polyethylene glycol dimethacrylate dimethacrylate, TEG‐GMA, 2.6 BHT and pigments	1. Apply composite resin in layers of no more than 2 mm thickness 2. Light cure for 20 s
MI Paste	Topical Paste	MI paste; GC Corp, Tokyo, Japan	Water, glycerol, CPP‐ACP, d‐Sorbitol, CMC‐Na, propylene glycol, silicon dioxide, titanium dioxide, Xylitol, phosphoric acid, flavoring, zinc oxide, sodium ethyl p‐sacrina, hidroxibenzoatol, magnesium oxide, guar gum, propyl p‐hydroxybenzoate, butyl p‐hydroxybenzoate.	Apply 0.1 mL of MI Paste actively using a brush for 3 min.

In this study, two different universal adhesive bonding systems were employed. The first system utilized was Gluma Bond Universal (Heraeus Kulzer GmbH, Hanau, Germany), while the second system used was All‐Bond Universal (Bisco, Schaumburg, IL, USA).

The prepared samples, a total of 160, were divided into four equal groups labeled A, B, C, and D, with each group containing 40 samples. These groups were categorized based on the pretreatment of the CAD surface. Each group was further divided into four subgroups (1, 2, 3, and 4) based on the type of universal adhesive system used (Gluma Bond Universal or All‐Bond Universal) and the mode of application (etch and rinse or self‐etch). Subgroups 1 and 2 utilized Gluma Bond Universal, while subgroups 3 and 4 used All‐Bond Universal. In subgroups 1 and 3, the universal adhesive systems were applied in E&R mode, while in subgroups 2 and 4, SE mode was employed.

For group A (control), the universal adhesive bonding systems were applied according to the manufacturer's instructions directly on the CAD surface without any prior pretreatment. In group B, a CPP‐ACP‐containing paste (MI paste; GC Corp, Tokyo, Japan) was actively applied using a brush for 3 min before applying the universal adhesive systems, following a thorough rinse with water. In group C, the CAD surfaces were pretreated with a PA solution for 1 min and then rinsed with water. The PA solution was prepared by dissolving 6.5 g of grape seed extract powder (Puritans Pride Inc., Oakdale, NY) in 100 ml of distilled water to create a 6.5% PA solution. In group D, the CAD surface was first pretreated with PA for 1 min, rinsed with water, and then CPP‐ACP paste was applied for 3 min. Following another rinse, the universal adhesive systems were applied.

In the E&R groups, the proanthocyanidin or CPP‐ACP was applied after the CAD surface was treated with 35% phosphoric acid gel (3 M, ESPE, St. Paul, MN) for 15 s.

**Figure 2:**
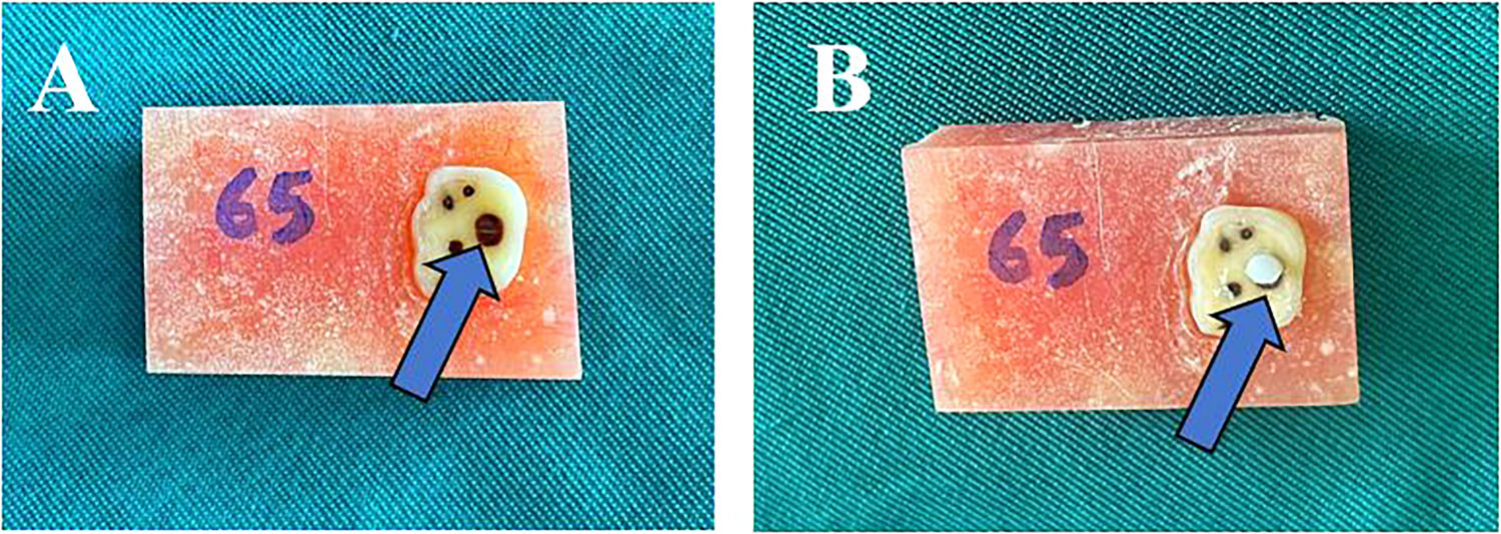
A prepared specimens before (A) and following (B) the bonding of composite resin. In (A), the flash points to the caries‐affected dentin. In (B), the flash highlights the composite cylinder bonded to the caries‐affected dentin.

To define the CAD surface, an adhesive tape with a punched hole at the center was used, and a translucent polyvinyl chloride microtube (0.7 mm internal diameter, approximately 0.5 mm height) was placed on it. The microtubes were then filled with Filtek Z350 XT composite resin (3M‐ESPE, St Paul, USA). Light curing was performed using a VIP Junior light curing unit (Bisco, Schaumburg, IL) at a power density of 600 mW/cm². A radiometer was used to verify and maintain consistent light intensity during the polymerization process. Figure 2 presents an illustrated depiction of a prepared bonded specimen.

### 2.3 Microshear Bond Strength and Failure Mode Analysis

**Figure 3:**
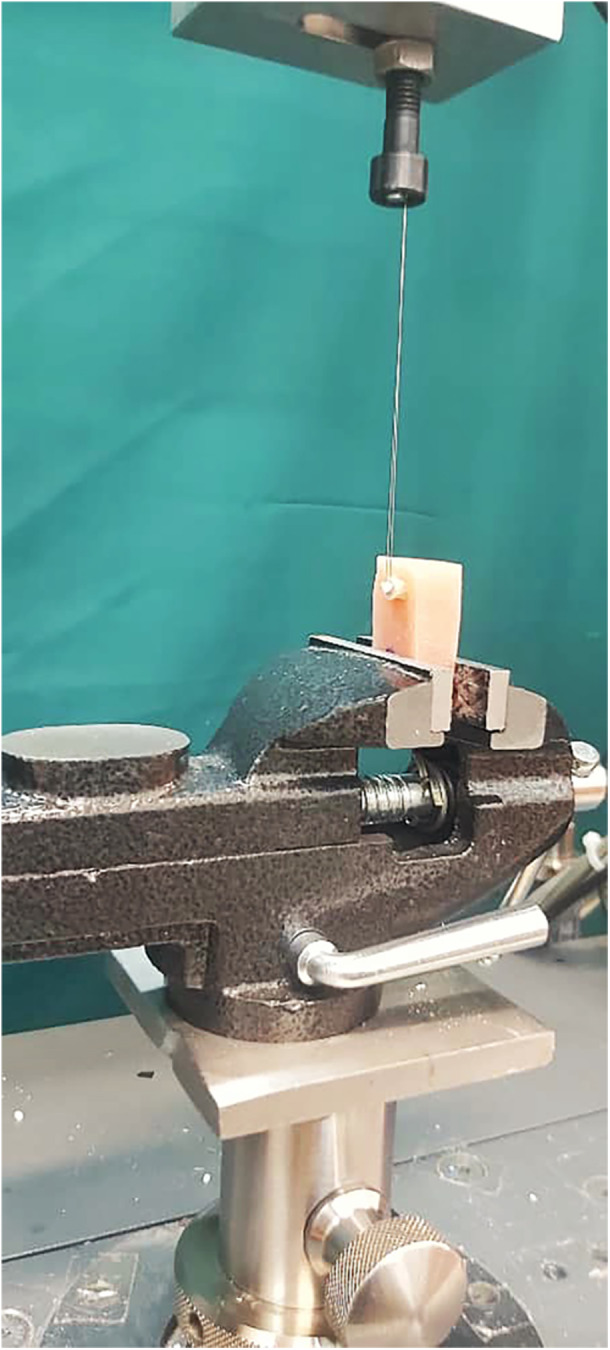
A bonded specimen where a stainless‐steel ligature wire is wrapped around the base of a composite resin micro‐cylinder, which is placed under the universal testing machine.

After 24 h of storage in distilled water at 37°C, the μSBS measurements were conducted on the specimens from each group (*n* = 10). A universal testing machine (Instron, Z020, Zwick Roell, Germany) with a crosshead speed of 1 mm/min was used, and the measurements were taken in a direction parallel to the bonded interface (Figure 3).

The failure mode analysis was conducted using a stereomicroscope at ×40 magnification (Carl Zeiss Inc., Oberkochen, Germany) and categorized into the following types:

A. Adhesive failure at the interface between the adhesive and composite, or at the interface between the adhesive and dentin (visible adhesive remaining on dentin), or

B. Cohesive failure within the composite or dentin, or

C. Mixed failure, which involves a combination of cohesive failure within dentin and/or composite, along with areas of adhesive failure (Jowkar et al. 2021).

To ensure consistency in failure mode classification, all evaluations were conducted by a single calibrated examiner. The examiner underwent prior training and repeated evaluations to ensure intra‐examiner reliability and to standardize the classification process. In cases where classification was uncertain, a second examiner was consulted, and a consensus was reached through discussion. This approach minimized subjective bias and enhanced the reliability of the failure mode assessment, aligning with established methodologies in failure mode analysis.

### 2.4 Statistical Analysis

The data distribution's normality was assessed using the Shapiro‐Wilk test. Subsequently, a three‐way analysis of variance (ANOVA) was conducted to examine the effects of three primary factors including the type of CAD pretreatment, the type of universal adhesive system employed, and the mode of application for the universal bonding systems. In case of significance, t‐test was applied. The Statistical Package for the Social Sciences (SPSS) software for Windows version 20.0 (IBM SPSS software; SPSS, Chicago, IL, USA) was utilized for data analysis. Statistical significance was determined at a significance level of *p* < 0.05.

## 3 Results

**Figure 4:**
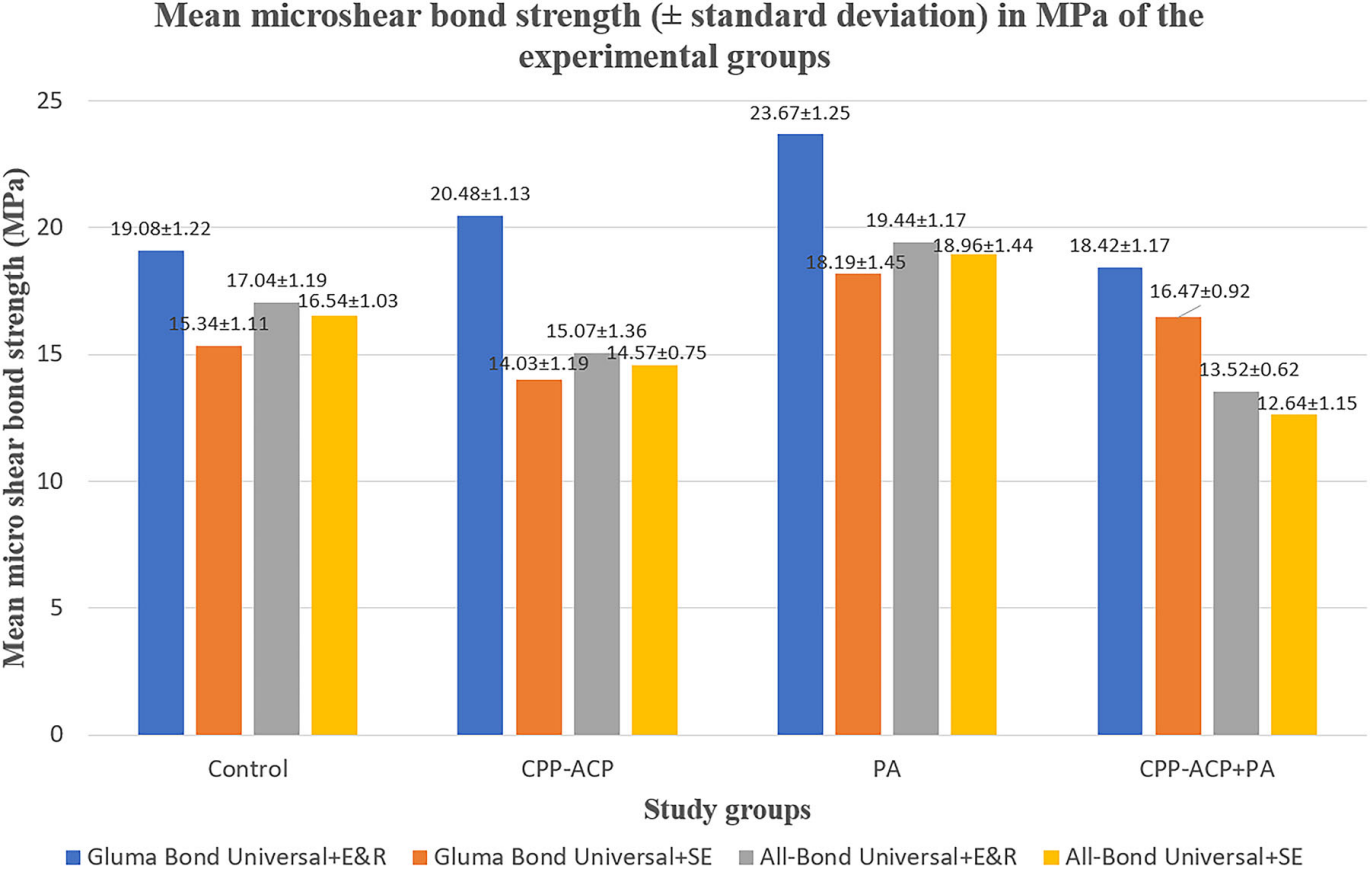
Bar chart depicting the microshear bond strength of the study groups. CPP‐ACP, casein phosphopeptide‐amorphous calcium phosphate paste; E&R, etch and rinse; PA, proanthocyanidin; SE, self‐etch.

Table 2 provides the mean μSBS values (including standard deviation, SD) in megapascals (MPa) for all study groups, while Figure 4 visually presents these values through a bar chart. The results of the three‐way ANOVA analysis, available in Table 3, revealed significant variations among the experimental groups (*p* < 0.001).

**Table 2:** Mean microshear bond strength (±standard deviation) in MPa of the experimental groups.

Group	Subgroup description	Mean microshear bond strength (±standard deviation)
A (control)	Gluma Bond Universal+ E&R	19.08 ± 1.22^a^
	Gluma Bond Universal + SE	15.34 ± 1.11^a^
	All‐Bond Universal+ E&R	17.04 ± 1.19^a^
	All‐Bond Universal+ SE	16.54 ± 1.03^a^
B (CPP‐ACP)	Gluma Bond Universal+ E&R	20.48 ± 1.13^ab^
	Gluma Bond Universal+ SE	14.03 ± 1.19^ab^
	All‐Bond Universal+ E&R	15.07 ± 1.36^ab^
	All‐Bond Universal+ SE	14.57 ± 0.75^ab^
C (PA)	Gluma Bond Universal+ E&R	23.67 ± 1.25^c^
	Gluma Bond Universal+ SE	18.19 ± 1.45^c^
	All‐Bond Universal+ E&R	19.44 ± 1.17^c^
	All‐Bond Universal+ SE	18.96 ± 1.44^c^
D (CPP‐ACP + PA)	Gluma Bond Universal+ E&R	18.42 ± 1.17^db^
	Gluma Bond Universal+ SE	16.47 ± 0.92^db^
	All‐Bond Universal+ E&R	13.52 ± 0.62^db^
	All‐Bond Universal+ SE	12.64 ± 1.15^db^

For each pretreatment group within the column, mean values with different lowercase superscript letters indicate statistically significant differences at a significance level of 0.05.

**Table 3:** Results of the three‐way ANOVA test.

Source	SS	df	Mean square	*F*	*p* value
Type of CAD pretreatment	531.325	3	177.108	131.761	< 0.001*
Type of universal adhesive bonding	200.077	1	200.077	148.849	< 0.001*
Mode of application of adhesive bonding	249.051	1	249.051	185.284	< 0.001*
Type of CAD pretreatment × Type of universal adhesive bonding system	81.428	3	27.143	20.193	< 0.001*
Type of CAD pretreatment × Type of universal adhesive bonding system	24.870	3	8.290	6.168	0.001*
Type of universal adhesive bonding system × Mode of application of adhesive bonding	145.161	1	145.161	107.994	< 0.001*
Type of CAD pretreatment × Type of universal adhesive bonding system × Mode of application of adhesive bonding	34.494	3	11.498	8.554	< 0.001*
Error	193.559	144	1.344	—	—
Total	48212.056	160	—	—	—

Abbreviations: ANOVA, analysis of variance; df, degrees of freedom; F, F statistic; SS, the sum of squares.

* Significant at *p* < 0.05.

The three‐way ANOVA highlighted the significant influence of the CAD pretreatment type on μSBS to CAD (*p* < 0.001). Subsequent one‐way ANOVA analysis revealed notable variations among different pretreatments (*p* = 0.014), prompting a post hoc Tukey test for further investigation. Results from the Tukey test underscored that CAD pretreatment with PA demonstrated the highest μSBS value (20.06 ± 2.50) in comparison to both the control group (no pretreatment, 17.00 ± 1.75) and other CAD pretreatments (p values < 0.001). Conversely, CAD pretreatment with CPP‐ACP + PA yielded lower μSBS (15.26 ± 2.52) than the control group (*p* = 0.009). No significant differences were observed between the CPP‐ACP group (16.03 ± 2.84) and the control group (*p* = 0.292), as well as between the CPP‐ACP group and the CPP‐ACP + PA group (*p* = 0.491).

Regarding the choice of universal adhesive bonding systems, the three‐way ANOVA revealed a significant impact on μSBS to CAD (*p* < 0.001). Further t‐test analysis unveiled noteworthy differences between the two types of universal adhesive bonding systems (*p* < 0.001), with Gluma Bond Universal exhibiting significantly higher bond strength than All Bond Universal.

Furthermore, the mode of application of the universal adhesive bonding system played a crucial role in μSBS to CAD, as indicated by the three‐way ANOVA (*p* < 0.001). Additional t‐test comparisons highlighted significant differences between the two application modes of the adhesive bonding systems. According to the t‐test results, the E&R mode resulted in significantly higher bond strength to CAD in primary teeth compared to the SE mode (*p* < 0.001).

**Figure 5:**
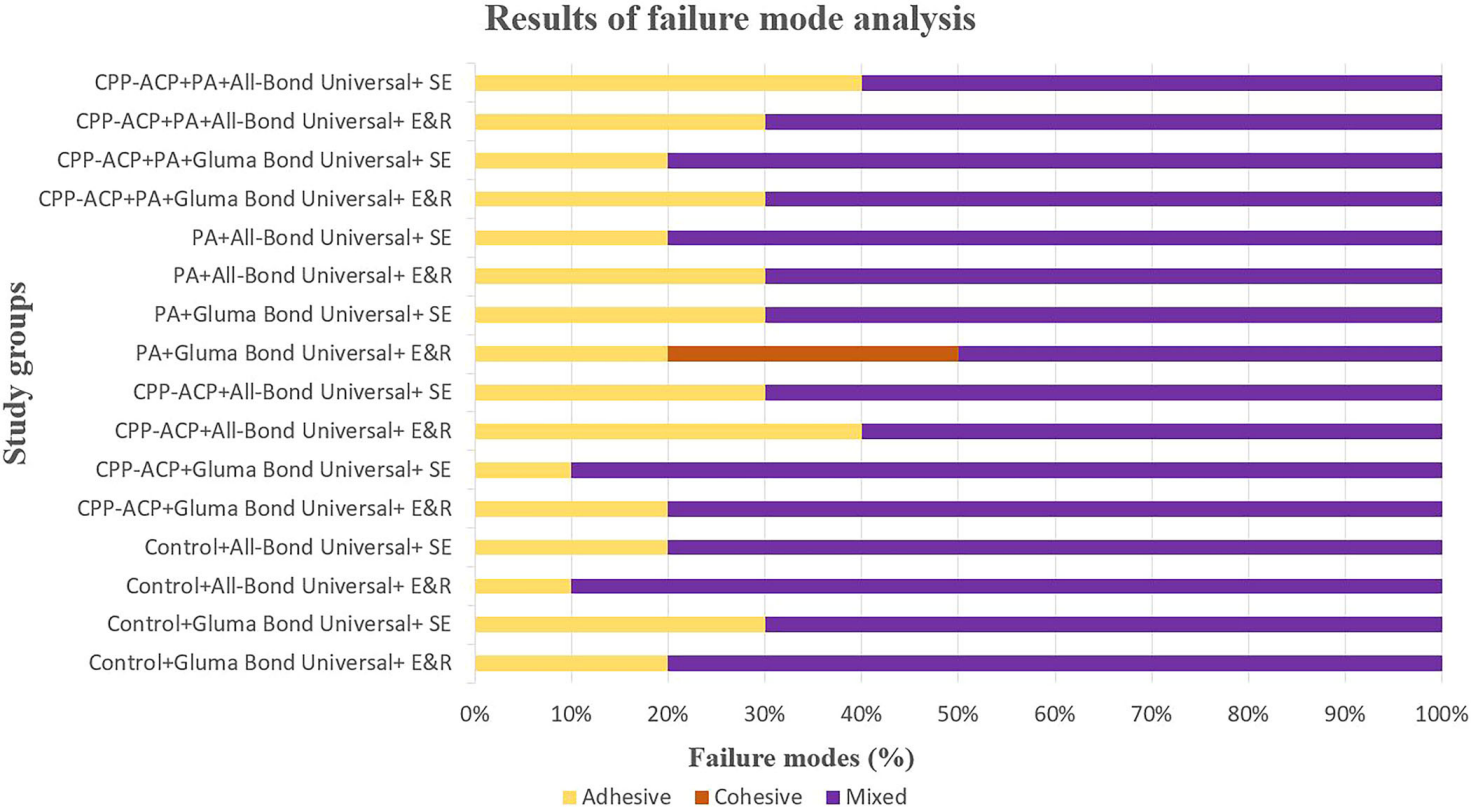
Bar chart illustrating the results of failure mode analysis. CPP‐ACP, casein phosphopeptide‐amorphous calcium phosphate paste; E&R, etch and rinse; PA, proanthocyanidin; SE, self‐etch.

**Figure 6:**
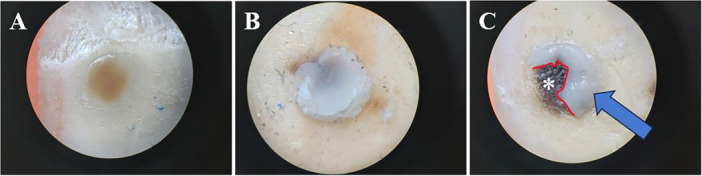
The stereomicroscope images (×40 magnification) showing different failure modes observed during the microshear bond strength test conducted in this study. (A) Adhesive failure, (B) Cohesive failure in the composite resin, and (C) Mixed failure with adhesive shown by “*” and arrows indicating the composite resin.

The results of failure mode analysis are presented in Figure 5, illustrating the various failure modes observed in the study. Among the study groups, mixed failure was found to be the predominant mode, observed consistently across all groups. In the PA + Gluma Bond Universal + E&R group, cohesive failure exclusively transpired in just three specimens within the composite resin. Additionally, Figure 6 showcases representative examples of different failure modes encountered during the μSBS test conducted in this study.

## 4 Discussion

The objective of this study was to evaluate the impact of CPP‐ACP and PA on the μSBS of universal adhesives to CAD in primary teeth. The rejection of the null hypothesis in our study indicates significant differences in bond strength outcomes. PA pretreatment showed the highest bond strength, surpassing CPP‐ACP and combined CPP‐ACP + PA pretreatments. Gluma Bond Universal outperformed All Bond Universal, emphasizing the importance of adhesive selection. The E&R mode yielded superior bond strength compared to SE.

The μSBS test is a reliable method for evaluating bond strength, particularly for small areas like CAD, as it allows multiple specimens from the same tooth. Unlike macroshear tests, which may cause uneven stress distribution and cohesive failure in dentin, the μSBS test minimizes these limitations (Sadat‐Shojai et al. 2010). Therefore, this study used the μSBS test to assess the effect of CPP‐ACP and PA on the bond strength of universal adhesives to CAD in primary teeth.

Adhesive efficacy varies between primary and permanent teeth due to structural differences. Primary teeth have thinner dental tissues, lower mineral content, and higher tubule density, leading to reduced intertubular dentin available for bonding (Nör et al. 1997; Shibata et al. 2016). Additionally, the peritubular dentin in primary teeth is significantly thicker, resulting in hybrid layers that are 25%–30% thicker than in permanent teeth (Ebrahimi et al. 2018).

Since CAD is often a significant part of cavity preparations, assessing bond strength is crucial for understanding its degradation. Structural changes in CAD, such as lower tensile strength, increased permeability, and larger acid‐resistant crystals blocking tubules, hinder adhesive infiltration (Wang et al. 2007). Additionally, exposed collagen fibrils and nanoleakage patterns compromise the hybrid layer, leading to reduced bond strength (Deyhle et al. 2011; Shibata et al. 2016). These factors contribute to early microleakage, recurrent caries, and pulpal irritation (Bahari et al. 2014). CAD also has a higher water content (14%–53%) than normal dentin ( ~10%), which affects adhesive performance (Nakajima et al. 2011). Enhancing mineral content at the dentin‐resin interface could improve mechanical properties and reduce hydrolysis. This study explores the potential of CPP‐ACP and PA in improving the μSBS of universal adhesives to CAD in primary teeth, which naturally have lower mineral content than permanent teeth (Ebrahimi et al. 2018).

As bacteria are absent in CAD, collagen cross‐linking stays intact, facilitating remineralization of intertubular dentin (Deyhle et al. 2011). An emerging approach to enhance CAD remineralization involves CPP‐ACP application, where CPP stabilizes ACP (Jowkar et al. 2021). CPP, derived from milk proteins, form clusters called CPP‐ACP by binding with amorphous calcium phosphate (Jowkar et al. 2021). This process promotes ion transfer, facilitating apatite crystal reconstruction (Bahari et al. 2014). CPP‐ACP not only enhances bond strength by increasing dentin mineral content but also helps prevent enamel subsurface demineralization (Reynolds 2009; Bahari et al. 2014). To achieve comprehensive remineralization, it's crucial to stabilize both the organic and inorganic components of the tooth, with CPP‐ACP primarily stabilizing the inorganic content (Bahari et al. 2014). Therefore, this study used PA, both alone and in combination with CPP‐ACP, for CAD pretreatment, aiming to create a more holistic remineralization approach by stabilizing both components.

Extracellular MMPs and cysteine cathepsins can degrade the organic matrix of conditioned dentin and the hybrid layer (Sultan et al. 2024). To counter this, external collagen cross‐linking agents have been explored to enhance dentin's resistance to enzymatic degradation (Sultan et al. 2024). Collagen cross‐linkers inhibit MMPs and cathepsins, improving the dentin/resin bonding interface (Vidal et al. 2014). PA, a bioflavonoid from plants like grape seeds, inhibits MMPs and cathepsin K, enhances collagen properties, and promotes crosslinking, strengthening the dentin collagen matrix (Bedran‐Russo et al. 2008). PA engages in hydrogen bonding with collagen fibrils, reduces enzyme binding sites, and lowers dentin hydrophilicity, which may limit resin penetration and reduce microleakage (Epasinghe et al. 2014; Beckman et al. 2024). Previous studies have shown that flavonoids like PA improve both immediate bonding performance and long‐term durability of universal bonding systems on CAD (Dávila‐Sánchez et al. 2020).

The study results revealed that using PA as a pretreatment for CAD resulted in the highest μSBS, whereas combining CPP‐ACP with PA showed lower μSBS compared to the control group. Interestingly, the bond strengths of the CPP‐ACP group were similar to those of the control group, indicating that CPP‐ACP application did not significantly enhance bond strength. These findings underscore the impact of various CAD pretreatments on bond strength and revealing the significance of selecting an optimal pretreatment strategy to improve bonding efficacy to CAD.

The present study aligns with previous research showing that PA pretreatment significantly enhances bond strength in both sound and CAD dentin (Degirmenci, Degirmenci et al. 2021). Another study also found that additional treatments, such as collagen cross‐linkers, improve bonding to CAD, especially with E&R adhesives (Isolan et al. 2015). PA demonstrated superior bond strength compared to CPP‐ACP due to its ability to cross‐link collagen fibers, improving the structural integrity and stability of dentin collagen (Bedran‐Russo et al. 2008). PA also protects collagen from enzymatic degradation and promotes remineralization by depositing calcium and phosphate, strengthening the tooth structure (Bedran‐Russo et al. 2008; Castellan et al. 2013). Furthermore, PA aids in collagen biosynthesis, benefiting CAD's remineralizable section (Bedran‐Russo et al. 2008). Unlike CPP‐ACP, which primarily stabilizes inorganic content, PA offers additional collagen stabilization and antioxidant effects, leading to stronger bonding. PA also facilitates the reexpansion of collapsed collagen fibrils during the drying phase, improving resin penetration and bonding. Further studies on the specific mechanisms of PA's enhanced bond strength compared to CPP‐ACP could offer valuable clinical insights.

The superior bond strength of PA alone over the CPP‐ACP + PA combination suggests that PA had a more pronounced effect on bond enhancement. PA likely strengthened the dentin collagen matrix through cross‐linking, whereas CPP‐ACP may have interfered with this process. The lack of a synergistic effect between PA and CPP‐ACP indicates potential interactions that diminished PA's efficacy. Additionally, mineral precipitation from CPP‐ACP might have limited PA's penetration, reducing its impact on deeper CAD regions.

Bahari et al. observed that the application of CPP‐ACP did not affect the microtensile bond strength of two adhesive systems (one E&R and one SE adhesive system) on CAD (Bahari et al. 2014). Similarly, Sattabanasuk et al. reported that CPP‐ACP application before a 3‐step E&R adhesive reduced bond strength due to residual precipitates blocking tubules and hindering resin infiltration (Sattabanasuk et al. 2009). The calcium phosphate from CPP‐ACP can close tubule orifices, potentially decreasing bond strength by obstructing adhesive penetration. While CPP‐ACP promotes remineralization, it may also block dentinal tubules, reducing permeability and limiting its remineralization benefits. Further research is needed to clarify CPP‐ACP's impact on CAD's mineral composition.

Efficient behavior management in pediatric dentistry relies on minimizing chair‐side time. Universal adhesives in SE mode offer a time‐saving alternative to traditional E&R systems, streamlining the application process (Lenzi et al. 2017). These adhesives, with less acidic monomers than phosphoric acid, help preserve the dentinal matrix and apatite crystals, which are particularly vulnerable to acid etching in primary teeth (Lenzi et al. 2017). By simultaneously demineralizing and infiltrating resin monomers, they contribute to maintaining dentin integrity and minimizing discrepancies and gap formation (Lenzi et al. 2017). A systematic review emphasized the role of adhesive pH in bonding efficacy, with structural and compositional variations affecting adhesive interfaces and performance (Cuevas‐Suarez 2019). Structural and concentration differences in these monomers lead to diverse adhesive interfaces, impacting bonding effectiveness (Wang et al. 2017). Therefore, this study compared the bonding effectiveness of All‐Bond Universal and Gluma Bond Universal, two adhesives differing in composition and pH, on CAD. The findings revealed that Gluma Bond outperformed All‐Bond Universal, regardless of surface pretreatment and application mode.

The superior performance of Gluma Bond may stem from its formulation, which effectively addresses challenges in bonding to CAD, including altered mineral content, increased porosity, and collagen degradation. With a pH of 1.8, Gluma Bond Universal is classified as an intermediary strong SE adhesive, offering better demineralization potential (Van Meerbeek et al. 2011). In contrast, All‐Bond Universal, with a pH of 3.2, falls into the ultra‐mild adhesive category (Choi et al. 2017). The higher efficacy of Gluma Bond Universal in this study may be due to the lower concentration of soluble calcium phosphate crystals in CAD, which requires stronger acidity for effective dissolution (Bahari et al. 2014). The increased acidity of Gluma Bond Universal likely enhances its ability to remove these mineral deposits from dentinal tubules, improving adhesive infiltration and bond strength.

All‐Bond Universal relies on 10‐MDP as its functional monomer, allowing partial dentin demineralization and submicron penetration (Hanabusa et al. 2012). 10‐MDP interacts with released calcium ions, forming stable 10‐MDP‐Ca salts within the hybrid layer, contributing to strong molecular adhesion based on the adhesion‐decalcification concept (Hanabusa et al. 2012). In contrast, Gluma Bond Universal contains both 10‐MDP and 4‐MET, which chemically interact with residual hydroxyapatite in dentin, forming stable 10‐MDP‐Ca or 4‐MET‐Ca salts that create hydrophobic nanolayers (Sanabe et al. 2009; Hanabusa et al. 2012). This reaction leads to the formation of a hydrophobic adhesive layer, which has superior mechanical properties and is more resistant to degradation compared to a hydrophilic layer (Sanabe et al. 2009). Gluma Bond Universal combines chemical bonding and micromechanical locking mechanisms to establish a hybrid layer (Sanabe et al. 2009). The combined chemical bonding and micromechanical interlocking of Gluma Bond Universal may explain its superior performance compared to All‐Bond Universal.

Gluma Bond Universal is an acetone‐based adhesive devoid of hydroxyethyl methacrylate (HEMA), while All‐Bond Universal is ethanol‐based and contains HEMA. A previous study investigated the bonded interface between an acetone‐based adhesive (Prime & Bond Elect; Dentsply Caulk; Milford, DE, USA) and healthy dentin, and no degradation of the hybrid layer was observed even after 1 year of aging (Zhang et al. 2016). Additionally, a separate study demonstrated that while All‐Bond Universal exhibited notable degradation when bonding to CAD, Prime & Bond Elect maintained consistent bond strengths at both 24 h and after 1 year (Follak et al. 2021). The acetone's higher vapor pressure might expedite solvent evaporation, reducing residual water retention compared to ethanol (Zhang et al. 2016). The improved performance of Gluma Bond Universal in bonding to CAD in the current study can be attributed to its acetone‐based composition, which, as suggested by previous findings, may enhance the stability and longevity of the adhesive interface.

This study found that universal adhesives in E&R mode consistently achieved higher bond strength than in SE mode, regardless of CAD pretreatment. This supports previous findings favoring E&R over SE for both sound and caries‐affected primary dentin (Thanaratikul et al. 2016). The superior performance of E&R adhesives on CAD may be due to their ability to dissolve acid‐resistant mineral casts and smear layers, improving resin infiltration and hybrid layer formation (Wang et al. 2007; Erhardt et al. 2008; Isolan et al. 2015; Shibata et al. 2016). Acid etching process allows for better mechanical interlocking with altered CAD, whereas SE adhesives, with their less acidic composition, have reduced potential to demineralize and create microporosities (Isolan et al. 2015). Therefore, etching facilitates deeper resin penetration into healthy dentin, leading to longer resin tags and thicker hybrid layers, which, as shown in a study, can contribute approximately 30% to the shear bond strength of an E&R adhesive (Gwinnett 1993; Thanaratikul et al. 2016). Moreover, the bonding mechanism of SE adhesives relies on chemical interaction with calcium ions, which are typically present in lower concentrations in CAD (Isolan et al. 2015).

Mixed failure was identified as the prevailing mode across all study groups, consistently observed throughout the experiments. However, it is noteworthy that within the PA + Gluma Bond Universal + E&R group, cohesive failure occurred exclusively in a small subset of three specimens within the composite resin. The exclusive occurrence of cohesive failure in the PA +  Gluma Bond Universal + E&R group can be attributed to its significantly higher bond strength compared to the other experimental groups. the higher bond strength observed in this group, which can be attributed to the strong bonding properties of Gluma Bond Universal and the application of the E&R technique. Additionally, the presence of PA in the E&R process may have further enhanced the bonding effectiveness.

This study chose to use CAD from natural lesions instead of artificial CAD due to histological differences (Isolan et al. 2015). Natural CAD, with features like transparent dentin, occluded tubules, and mineral deposits, forms over a longer period and may affect dentin behavior differently than artificial CAD (Isolan et al. 2015). Therefore, utilizing natural CAD in the present study allows for a more accurate representation of the complex nature of CAD compared to artificial CAD.

In this study, it was found that PA pretreatment showed the highest bond strength, surpassing CPP‐ACP alone or combined with PA. Gluma Bond Universal outperformed All Bond Universal, highlighting the importance of adhesive selection for optimal bonding on CAD in primary teeth. The E&R mode also demonstrated better bond strength than the SE mode, emphasizing the importance of correct application techniques in pediatric restorations.

It is important to acknowledge the limitations of this study. Firstly, the investigation was restricted to evaluating only two commercially available universal adhesives. Future studies should aim to include a more diverse selection of universal adhesive systems with varying pH levels. Additionally, this study solely focused on immediate bond strengths, measured after a 24‐h water storage period, which does not provide insight into the long‐term durability of the bonds. While immediate bond strength is a crucial predictor of initial adhesive performance, it should be mentioned that aging studies, such as thermocycling or prolonged water storage, would provide a more comprehensive understanding of long‐term bond stability. Therefore, future research should incorporate artificial aging protocols and clinical trials to assess adhesive performance over extended periods in real‐world scenarios. Furthermore, to complement the quantitative bond strength analysis, scanning electron microscopy should be employed in future studies to provide a more detailed evaluation of failure modes and interfacial characteristics. Additional laboratory experiments and randomized clinical trials with longer follow‐up durations are also necessary to validate and strengthen the findings of this study.

## 5 Conclusion

In conclusion, the study underscores the crucial role of CAD pretreatment, particularly with PA, in influencing μSBS in primary teeth. PA emerged as a standout pretreatment, exhibiting the highest μSBS compared to other methods, emphasizing its potential in fortifying bond strength in pediatric dentistry. Optimal outcomes were achieved with Gluma Bond Universal and the E&R method, underscoring the significance of adhesive selection and application technique in CAD scenarios. These findings offer valuable insights for refining adhesive protocols, potentially enhancing clinical outcomes and patient satisfaction in restorative procedures for primary teeth affected by caries. By integrating Proanthocyanidin, Gluma Bond Universal, and the E&R method into practice, clinicians can elevate treatment efficacy and the quality of care in pediatric dental settings, effectively addressing the challenges posed by CAD.

## Author Contributions

Zahra Jowkar and Ali Nozari conceived the ideas. All authors collected the data. All authors analyzed the data. Z. Zahra and Ali Nozari led the writing.

## Conflicts of Interest

The authors declare no conflicts of interest.

## Data Availability

Data is available on request from the author.
